# Characterization of geological and lithological features in the area proximal to tritium-contaminated groundwater at the Semipalatinsk test site

**DOI:** 10.1371/journal.pone.0300971

**Published:** 2024-03-22

**Authors:** Medet Aktayev, Sergey Subbotin, Assan Aidarkhanov, Almira Aidarkhanova, Lyubov Timonova, Natalya Larionova

**Affiliations:** 1 Institute of Radiation Safety and Ecology, National Nuclear Center of the Republic of Kazakhstan, Kurchatov, Kazakhstan; 2 L.N. Gumilyov Eurasian National University, Astana, Kazakhstan; Universiti Teknologi Malaysia, MALAYSIA

## Abstract

The article presents the results of a study of groundwater contaminated with tritium in the vicinity of the ‘Atomic Lake’ - a crater filled with water as a result of a thermonuclear explosion on the territory of the former Semipalatinsk test site. This crater was created as part of an experimental thermonuclear explosion in 1965 with the aim of creating an artificial reservoir in arid areas. The study was carried out to identify the source of groundwater contamination near the crater formed from a thermonuclear test. There were two possible factors of pollution: the influence of contaminated water from the crater on the groundwater of the adjacent area, or groundwater polluting the water in the crater. It was necessary to find out the source of groundwater contamination and its connection with the water in the funnel. For this purpose, a study of the geological and lithological conditions of the territory adjacent to the funnel was carried out, which was carried out using drilling operations and hydrological measurements. Drilling work made it possible to study the depth of distribution of groundwater, hydrological work made it possible to determine the conditions of distribution of groundwater, as well as to take samples of groundwater. The assessment of the degree of groundwater contamination was carried out through water sampling and laboratory analysis. As a result, it was established that the geological and lithological conditions of the area limit the flow of contaminated groundwater to the water in the crater - the ‘Atomic Lake’. Despite the fact that the waters in the crater from a thermonuclear explosion and the groundwater of the adjacent territory are contaminated with the radionuclide tritium, they have different sources of contamination and are not interconnected. Radionuclide analysis of groundwater showed that increased concentrations of tritium with a specific activity of up to 95 000 Bq/l are found in groundwater near the river bed. Shagan and this is due to the influence of the flow of groundwater coming from other parts of the landfill.

## Introduction

It is known that the sources and routes of entry of induced radionuclides into surface and groundwater differ and are unique in their own way. One of the most complex and insufficiently studied processes is entry of radionuclides into groundwater and their migration in geological formations [1, 2]. The main sources of groundwater pollution includes nuclear tests, various accidents at nuclear fuel cycles (Chernobyl, Fukushima-1, Kyshtymsk accident, etc.), as well as leaks from the underground radioactive waste depositories [3–5]. Different radionuclides behave differently in groundwater and geological environments, depending on the physicochemical factors. Notably, among all existing radionuclides, tritium, having high migration capacity, is an indicator of the dynamics of transportation by groundwater [6–10].

**Fig 1:**
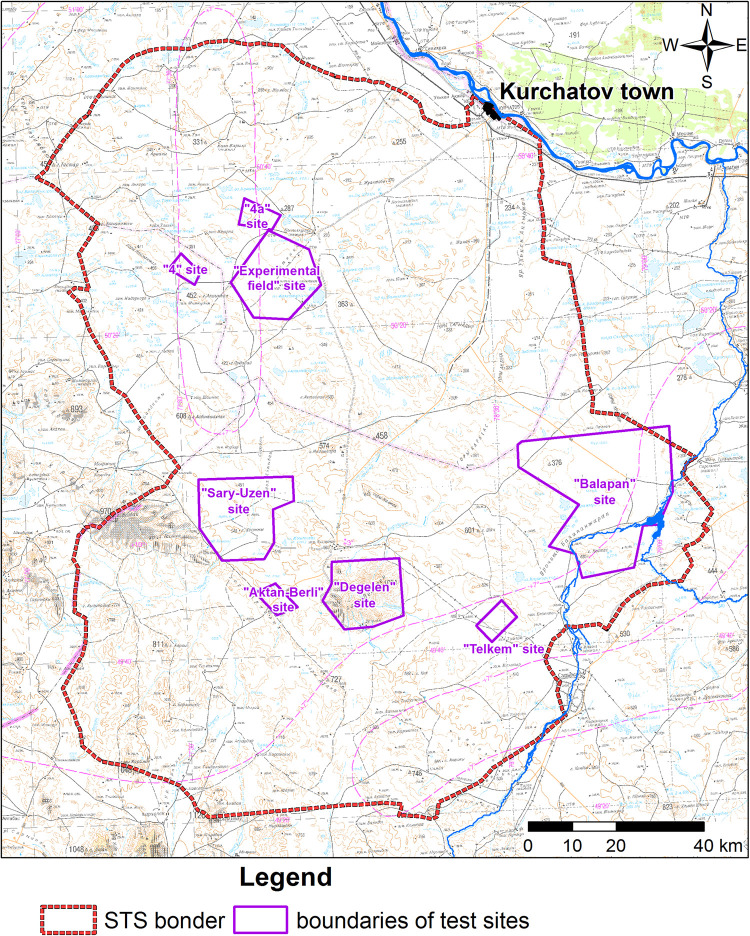
Territory of the former Semipalatinsk test site.

The examination of radionuclide migration by groundwater is of particular relevance and is one of the priorities after the closure of the Semipalatinsk Test Site (STS) [11–13], where over 456 nuclear tests were performed between 1949 and 1989, for both military and peaceful purposes (Fig 1).

**Fig 2:**
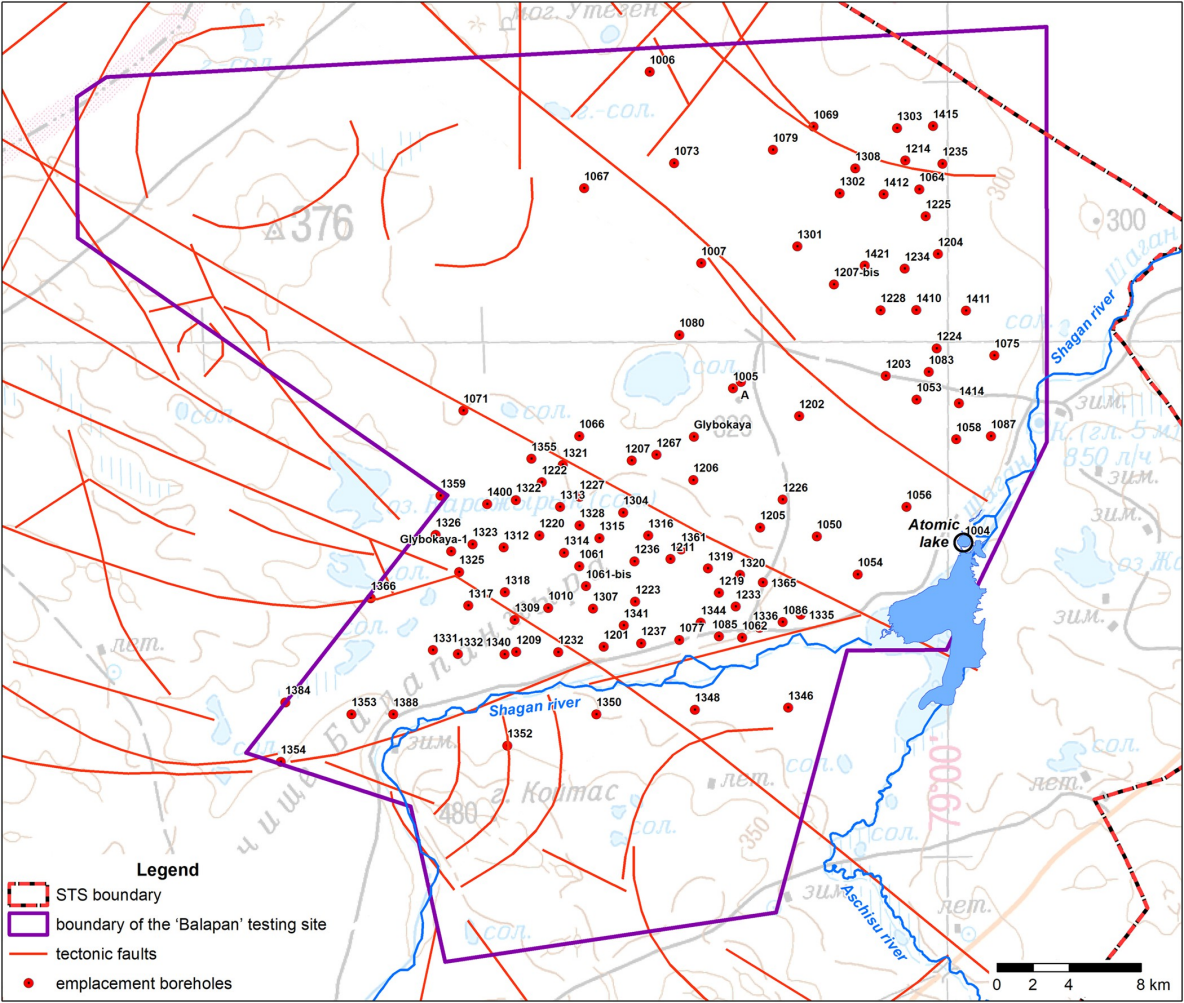
Balapan site territory.

One of the STS test grounds for underground nuclear weapons testing was the Balapan site located in its eastern part (Fig 2).

A total of 105 nuclear tests were performed at the Balapan test site from 1963 to 1989, including an excavation explosion with soil discharge as a part of the experiment to create an artificial reservoir [14]. The excavation explosion was carried out in 1965 in borehole No. 1004. This experiment was the first pilot industrial to obtain information on the possibility of using nuclear charges to create reservoirs in arid areas of the former USSR, in particularly Kazakhstan. As the result of explosion, a funnel with diameter of about 400 m and depth of 100 m was created at the junction of the Shagan and Aschisu rivers [15, 16]. After the explosion, the valleys of the Shagan and Aschisu rivers was connected to the funnel to allow river water to pass through. This created conditions for the formation of two bodies of water: an internal and external one. The water-filled inner funnel was later named the ‘Atomic Lake’ (Fig 2).

Long-lived radionuclides were developed from the excavation explosion, which concentrated in the soils surrounding the ‘Atomic Lake’ [17]. The specific activity of induced radionuclides is 4 000 Bq/kg at ^241^Am, 15 000 Bq/kg at ^137^Cs, 17 000 Bq/kg at ^239+240^Pu, 10 000 Bq/kg at ^90^Sr, 65 000 Bq/kg at ^3^H, 20 000 Bq/kg at ^152^Eu, and 13 000 Bq/kg at ^154^Eu [18]. Despite the high radionuclide contents in the piling soils, radionuclides such as ^137^Cs, ^241^Am, ^239+240^Pu and ^152^Eu are practically not detected in the lake water. The content of ^3^H in water ranges from 100 to 20 000 Bq/l, and the specific activity of ^90^Sr varies from 1 to 20 Bq/l [19, 20].

Currently, migration of induced radionuclides with groundwater from the ‘Balapan’ site is considered one of the sources of water pollution of the ‘Atomic Lake’ and adjacent territory [21]. The reason is that earlier the site for unloading tritium polluted groundwater was found 4-5 km downstream the Shagan river [22]. It was found that on the left bank of the Shagan River, the water-resistant clays of the ‘Balapan’ site are wedged into the floodplain of the river, thereby unloading polluted waters through the channels of fissure distortions [23, 24]

**Fig 3:**
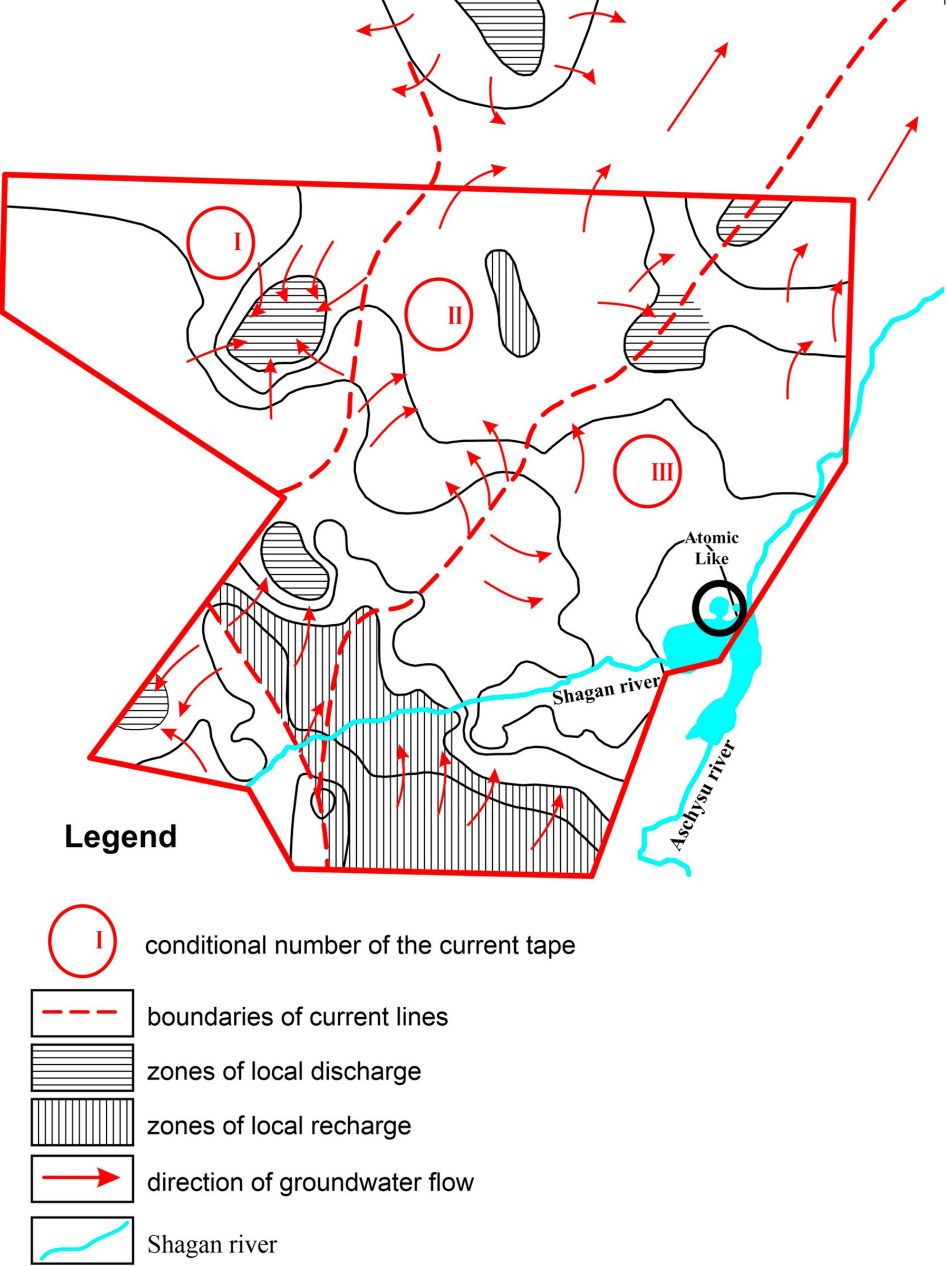
Structure of groundwater filtration flow.

The entry of tritium-contaminated ground waters beyond the Balapan site is directly related to the feature of how streams move in the structure of run bands (Fig 3).

In the northwestern section (band No. 1), the filtration flow is formed at high levels of the Paleozoic basement. The direction of groundwater flow is limited by local depression. In the central area, local watershed No. 2 can be noted. However, this area is also limited for the flow of groundwater by a depression. Beyond the northern boundary of the site, the relative height of the aquifer topography increases. In this regard, the flow changes its direction towards strip No. 3. Within this strip there are more than 50 “combat” wells, from which the flow of groundwater can flow into the territory of the studied object.

In general, the groundwater common in the Balapan site is divided into fissure and pore depending on the geological conditions. Fissure waters may directly enter the surface flow or lake, in the absence of overlying water-resistant rocks, or, in the presence of overlying friable sedimentary rock, mix with groundwater and flow into the river or lake in this form. Groundwater, if not connected to fissure waters, can flow directly into the surface flow.

Thus, the purpose of this work was to determine the sources of groundwater pollution in the area of the crater, as well as to investigate the likelihood of tritium-contaminated groundwater entering the ‘Atomic Lake’ through geological channels from the Balapan site.

## Materials and methods

The study area is one of the areas insufficiently provided with precipitation. Annual precipitation varies from 77.0 mm to 362.0 mm. The maximum amount of precipitation was in 2010–900.0 mm/year, the minimum - in 2013 (77.0 mm/year). According to the relief, there are low mountains, high and low small hills, deluvial-proluvial inter-hill plains, alluvial plains of river valleys and temporary watercourses, lake and saline depressions. The maximum height of 320 m above sea level is noted on the crest of the ‘Atomic Lake’ funnel; in flat areas of the territory it is 280 m.

Well drilling, water sampling and laboratory analysis works were carried out to examine the geological and lithological conditions and tritium pollution of groundwater.

### Research sites

**Fig 4:**
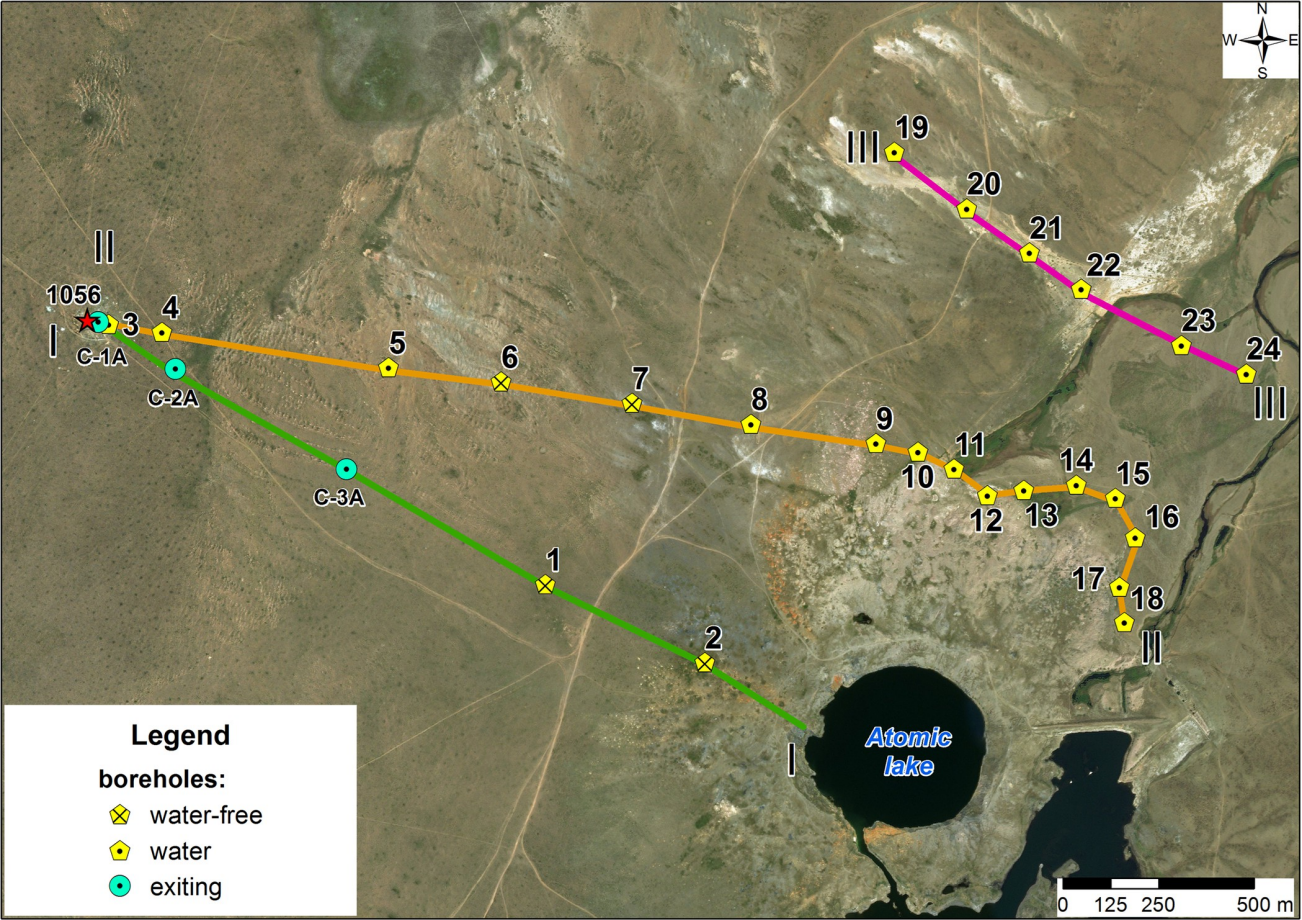
Groundwater examination area.

Three research profiles have been laid in order to identify possible routes of entry of tritium-polluted groundwater. Two profiles along the line I-I and II-II have been laid starting from the nearest ‘emplacement’ borehole 1056 of the Balapan site in the direction of the ‘Atomic Lake’ and exits of the Shagan river from the ‘Atomic Lake’. The third profile on line III-III runs from the side of the Balapan site and crosses the old bed of the Shagan river from the left bank 1 km away from the ‘Atomic Lake’ (Fig 4).

### Borehole drilling

Three existing boreholes S-1A, S-2A and S-3A were located along the profile I-I line. In other cases, 24 new holes were drilled. Boreholes were drilled using the KamAZ chassis based LBU 50-07 drilling rig and the KV 10/10P compressor. Two drilling methods were used: mechanical rotational method for friable sedimentary rock and a rotary-percussive drilling method for hard rocks.

### Geological and lithological research

During the drilling process, a description of lithological layer and rock content on each well was performed. At the end of the drilling works, the downhole and groundwater level were measured in all boreholes. All records were entered in the drilling log. Further, in the office environment, construction of geological sections was carried out using a graphical editor. Rock designations are in compliance with GOST 21.302-2013.

### Sampling

Sampling was carried out by lowering the sampler into the borehole to a depth below groundwater level. The sample volume was calculated based on the amount of water required for radionuclide analysis and was 0.5 L.

### Laboratory analysis

To determine specific activity of tritium, preliminary preparation of selected water samples was carried out, namely filtration and subsequent mixing of 5 ml of distillate sample aliquot with scintillation cocktail in 1:4 proportion. The specific activity of tritium was determined by liquid scintillation spectrometry on ‘TRI-CARB 2900 TR’ (‘PerkinElmer’, USA) β-spectrometer. The error in determining the specific activity of tritium in water samples was ± 10%. The limit of detection for minimally detectable tritium activity was <6 Bq/l.

### Processing of results

The specific activity of ^3^Н in water was determined by the standard formula:

$$A\;in\;water=\frac{CPM-CPM\;scint}{Vsample∙60∙Eff}∙1\;000,\;\mathrm{Bq}/l$$

where:

CPM - number of pulses per minute registered by the spectrometric equipment ‘TRI-CARB 2900 TR’ for the measured sample, imp/min;

CPMscint - number of pulses per minute, registered by spectrometric equipment ‘TRI-CARB 2900 TR’ for ‘Ultima Gold’ scintillator, imp/min;

Vsample - volume of the measured sample (5 ml);

60 - conversion from minutes to seconds (from imp/min to imp/s), since 1 Bq is one decay per second;

1 000 - conversion of the value from ml (mg) to l (kg);

Eff – ^3^Н registration efficiency, determined by the formula:

$$Eff=\frac{CPM-CPM\;scint}{Aknown∙60}$$

where:

Aknown - known activity of the calibration ^3^Н source, Bq;

The minimum detectable activity (MDA) for the determination of ^3^H in water was calculated by the formula:

$$MDA=2\cdot \frac{\surd (CPMfon\cdot t)}{t∙60∙Eff\cdot Vsample}\cdot 1\;000,\;\mathrm{Bq}/l$$

where:

CPMfon - number of pulses per minute registered by spectrometric equipment ‘TRI-CARB 2900 TR’ for pure distilled water, imp/min;

t - measurement time, min.

## Study results

### Area on I-I line

**Fig 5:**
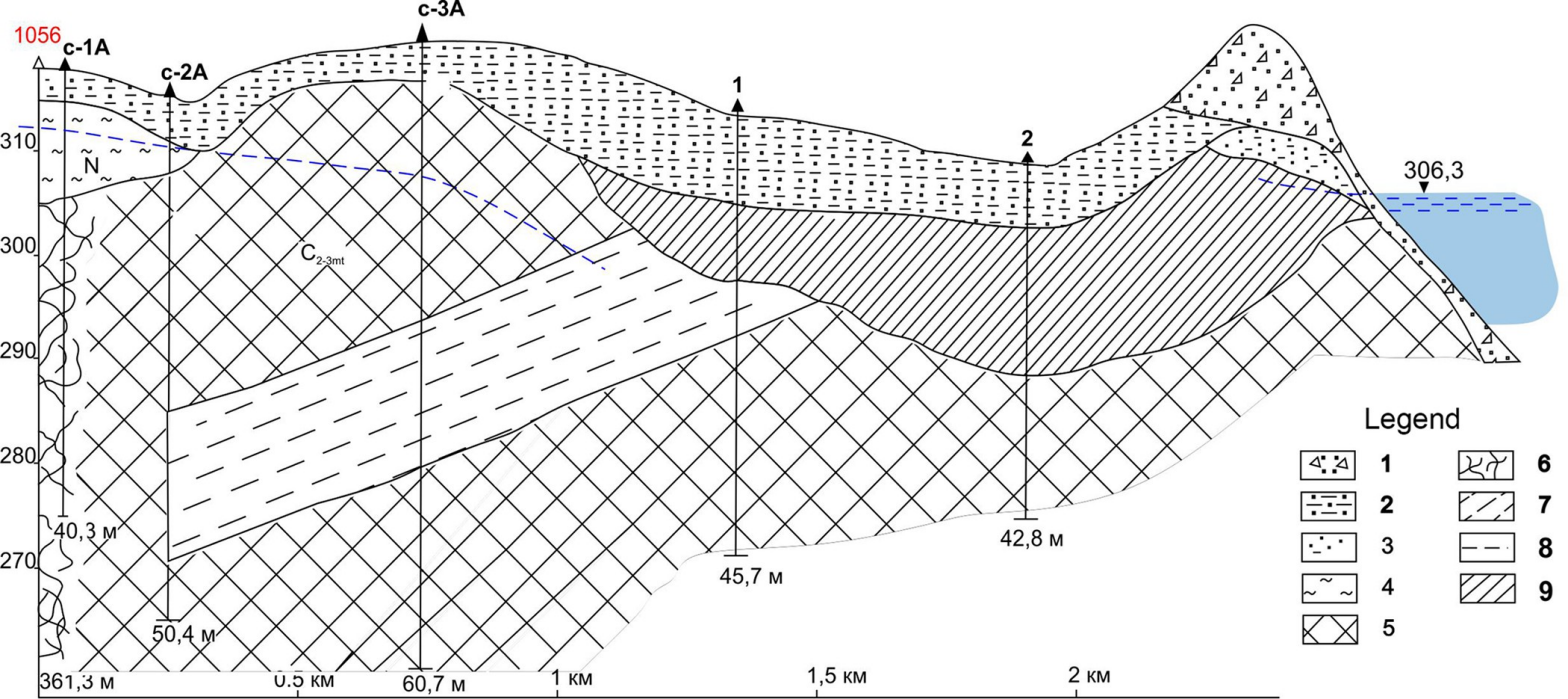
Geological section at I-I line.

Based on the drilling results, deluvial and proluvial sandy loams and sands, terrigenous layer of middle-upper carbonic are mainly developed in the battery of boreholes made. In the upper part of the section, rocks are subject to exogenous weathering. During drilling in boreholes 1 and 2 it was not possible to tap the aquifer. Geological section was built based on the results (Fig 5).

Results of laboratory test for determination of tritium specific activity in groundwater are presented in table (Table 1).

**Table 1:** Results of drilling and tritium analysis in groundwater.

Item No.	Borehole No.	B/h depth, m	Groundwater level, m	^3^H, Bq/l
1	S-1A	43.0	5.7	280 000 ± 28 000
2	S-2A	50.4	4.6	15 000 ± 1 500
3	S-3A	60.7	13.0	20 ± 2
4	1	45.7	no water detected	-
5	2	42.8	no water detected	-

*- laboratory analysis not performed

According to the data presented, maximum specific activity of tritium 280 000 Bq/l was detected in the S-1A borehole located 60 m away from ‘emplacement’ borehole 1056. Further, in the S-2A borehole at a distance of 200 m towards the ‘Atomic Lake’, there is a sharp decrease in specific activity of tritium to 15 000 Bq/l and to 20 Bq/l in the S-3A borehole at a distance of 800 m. So, the specific activity of tritium decreases to a minimum as moving away from the ‘emplacement’ borehole. This indicates that contaminated groundwater is localized only within the rock collapse zone in well 1056.

Absence of water in boreholes 1 and 2 most likely stems from the block of rocks (shales and siltstones) opened by borehole S-3A obstructing movement of groundwater towards the ‘Atomic Lake’.

### Area on II-II line

In this area, according to lithological descriptions, the upper part of the sections (boreholes 3-5) contains silt sandy loam, fine-grained sand with gravel in it. Groundwater levels in boreholes 3, 4 and 5 vary from 5 to 9 m. Water resistant deposits show beginning of stiff clays, which restrict water flows towards the bed of the Shagan river, hence underground water in borehole 6 and 7 was not detected. Next, in borehole 8, layer of groundwater in the form of a lens is detected in clay loam with sand.

**Fig 6:**
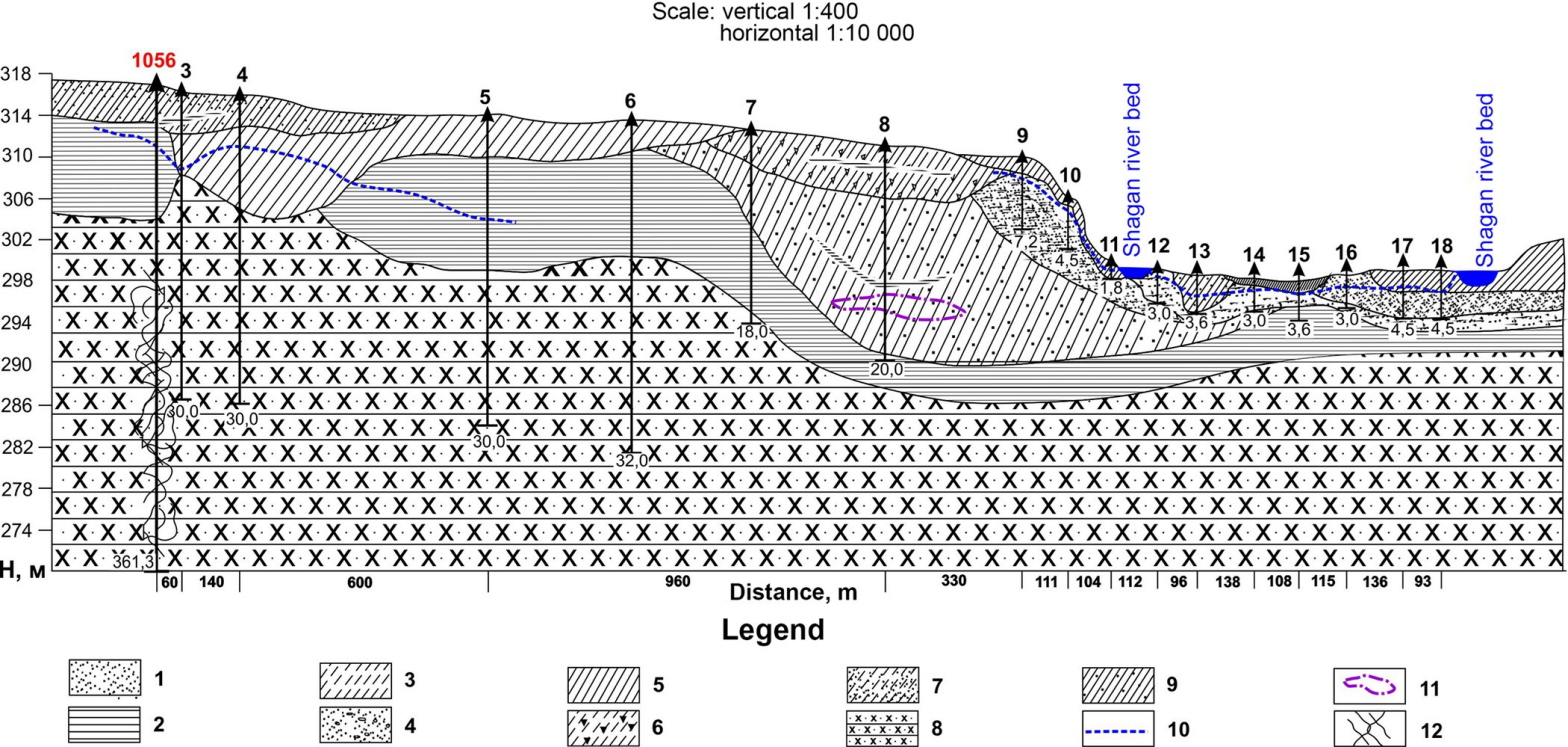
Geological section at II-II line.

At the bottom of the section (boreholes 9-18), the aquifer contains by multi-grained sand with gravel mixed in it with layers of clay loam. The maximum level is detected at 2.5 m in borehole 9 – the area of the terrace above flood-plain. The minimum level is set at 0.7 m in borehole 11 where the Shagan river exits from under a funnel piling. Geological section was constructed based on the results of the description of the lithological composition (Fig 6).

Results of laboratory test for determination specific activity of tritium in groundwater are presented in table (Table 2).

**Table 2:** Results of drilling and tritium analysis in groundwater.

Item No.	Borehole No.	B/h depth, m	Groundwater level, m	^3^H, Bq/l
1	3	30	8.0	37 000 ± 3 700
2	4	30	5.0	8 000 ± 800
3	5	30	9.0	<6
4	6	30	No water detected	-
5	7	30	No water detected	-
6	8	20	0.7	<6
7	9	7.2	2.5	20 000 ± 2 000
8	10	4.5	1.0	45 000 ± 4 500
9	11	1.8	1.0	95 000 ± 9 500
10	12	3.0	0.7	8 500 ± 8 500
11	13	3.6	2.0	5 500 ± 550
12	14	3.0	1.0	6 500 ± 650
13	15	3.6	1.2	5 500 ± 550
14	16	3.0	1.0	4 000 ± 400
15	17	4.5	1.5	3 000 ± 300
16	18	4.5	2.0	700 ± 70

*- laboratory analysis not performed

The results showed that specific activity of tritium decreases as moving away from the ‘emplacement’ borehole 1056. For example, in borehole 3 located 60 m from the ‘emplacement’ borehole, specific activity of tritium was 37 000 Bq/l, in 140 m in borehole 4, the content decreased to 8 000 Bq/l, and in borehole 5 located in 600 m the specific activity of tritium decreased to below the detection limit (<6 Bq/l).

Thus, we can state that in the Profile II-II area, polluted groundwater from the ‘emplacement’ borehole 1056 do not reach the Shagan river valley. This is evidenced by the presence of stiff seat clays in boreholes 5, 6 and 7, which form a watershed between groundwater in boreholes 8, 9, 10 and 11.

Specific activity of tritium at the bottom part of the section from 700 to 95 000 Bq/l (boreholes 9-18) increase because these boreholes are drilled in the offshore area of the Shagan river bed, and are located in the area of influence (recharge) of polluted river water coming out of the piling of the ‘Atomic Lake’ soils.

### Area III-III

**Fig 7:**
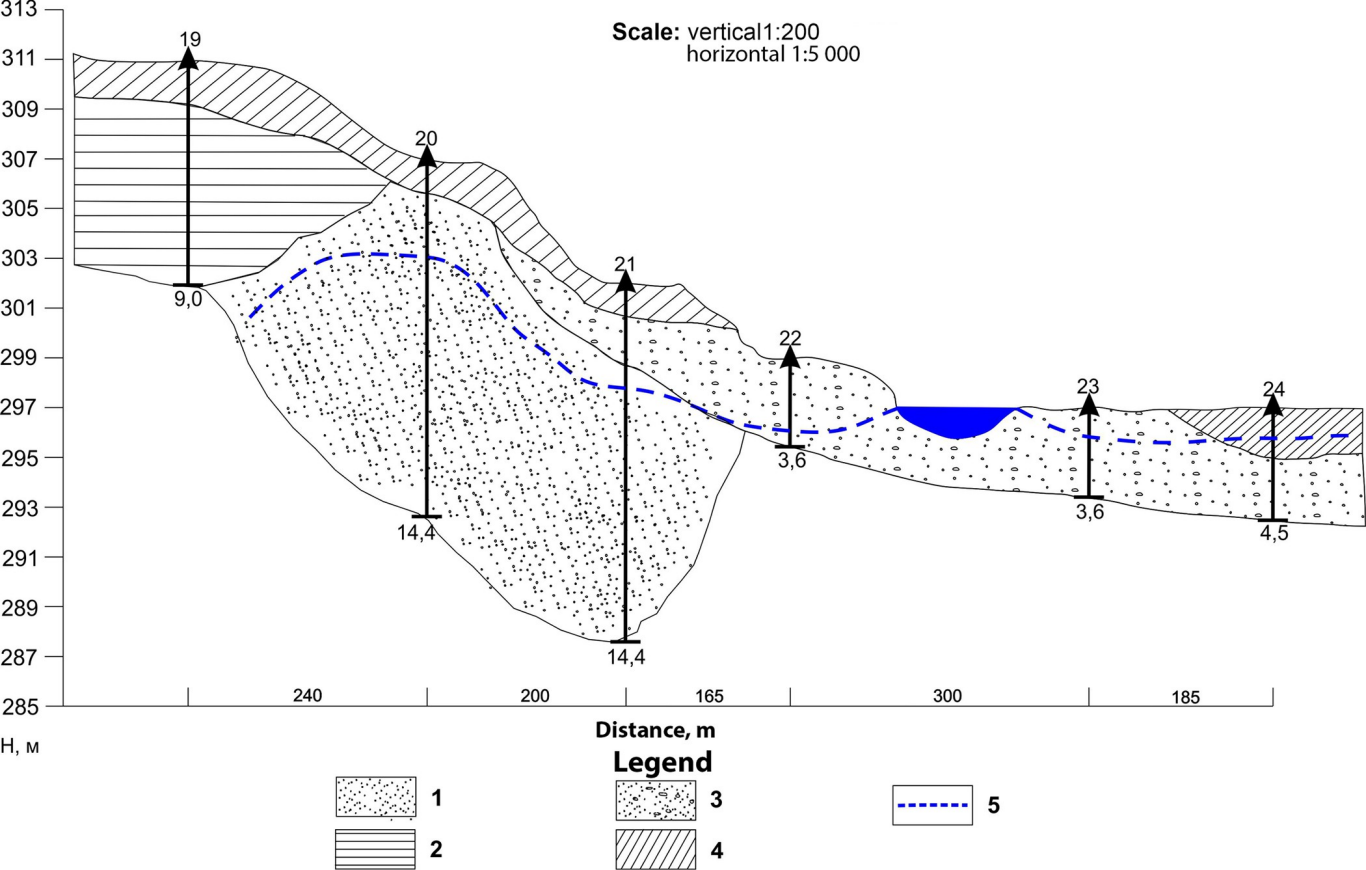
Geological section at III-III line.

Based on the results of drilling, the top of the lithological layer contains dry silt loams. No groundwater was detected in borehole 19. This is probably due to stiff clays that limit supply of groundwater from the Balapan site. The aquifer was tapped from borehole 20 at depth of 1.2 to 4.3 m and was characterized by layers of fine sands with admixed gravel (Fig 7).

Results of laboratory test for determination specific activity of tritium in groundwater are presented in table (Table 3).

**Table 3:** Results of drilling and tritium analysis in groundwater.

Item No.	Borehole No.	B/h depth, m	Groundwater level, m	^3^H, Bq/l
1	19	4.5	no water detected	-
2	20	14.4	4.0	<6
3	21	14.4	4.3	<6
4	22	3.6	3.0	15 000 ± 1 500
5	23	3.6	1.2	7 500 ± 750
6	24	4.5	1.2	5 500 ± 550

*- laboratory analysis not performed

Based on laboratory tests in groundwater of boreholes 20 and 21, the specific activity of tritium is below the detection limit of hardware and methodological support (<6 Bq/l). Specific activity of tritium then varies from 5 500 to 15 000 Bq/l in boreholes 22, 23 and 24.

Absence of groundwater in borehole 19 stems from presence of waterproof clays, which limits the groundwater flows from the Balapan site. This is also confirmed by absence of tritium in the groundwater of boreholes 20 and 21.

The increased specific activity of tritium in the groundwater of boreholes 22, 23 and 24 are attributed to influence of the Shagan river bed, as these boreholes are drilled in floodplains of the river.

## The discussion of the results

Thus, based upon drilling outputs, two types of ground waters have been uncovered in the study section – fracture and pore water.

According to the data obtained along the I-I profile line, groundwater contamination in this area is associated with the influence of radioactivity from well 1056. At the same time, flows of contaminated groundwater in this direction do not flow towards the ‘Atomic Lake’ funnel.

In section II-II, pore waters are associated with fractured waters and represent a single hydrogeological aquifer. In this case, radioactive contamination of pore waters is associated with the influx of tritium-contaminated fracture waters flowing from the regional basin. The second type of pore water was identified by wells No. 9–18 along descent II–II and wells No. 20–24 along descent III–III. These are pore waters of alluvial-proluvial deposits, including subbottom waters of the river Shagan. Radioactive contamination of this type of water is associated with the seepage of water flow through the dump of contaminated rocks of the ‘Atomic Lake’. The second type of pore water in areas II-II and III-III belongs to a single stream of the river Shagan underwater waters.

## Conclusions

Groundwater from the Balapan site does not affect the level of tritium pollution of the ‘Atomic Lake’ waters and the Shagan river waters around the exit from the lake (up to 1 km). This is confirmed by the geological and lithological characteristics and hydrogeological conditions of the area. In particular, absence of groundwater in a number of drilled boreholes and presence of waterproof clays and rocks limiting the spread of groundwater from the ‘emplacement’ borehole 1056 towards the ‘Atomic Lake’

Radionuclide analysis of groundwater in all studied profiles showed that in areas located near the Balapan site, the tritium concentration is below the detection limit (<6 Bq/l), with the exception of groundwater contamination along profile I-I. The source of groundwater pollution in this area is well 1056. Also, numerical concentrations of tritium with a specific activity of up to 95 000 Bq/l are found in wells located near the river bed. Shagan and navala of the ‘Atomic Lake’. The source of pollution in these areas is associated with the influence of the river Shagan, which washes out tritium when filtered through the ‘Atomic Lake’ funnel.

Thus, the flow of groundwater contaminated with tritium through geological channels from the Balapan site into the ‘Atomic Lake’ does not occur.
